# Outpatient Rescue With Stem Cells Boost for Refractory Hematotoxicity Following anti‐BCMA CAR T‐Cell Therapy: A Case Report

**DOI:** 10.1155/crh/1558475

**Published:** 2026-06-04

**Authors:** Mehdi Loukhnati, Benjamin Hebraud, Marine Cazaux, Cécile Borel, Pierre Bories, Jean Alain Martignoles, Miguel Granell, Pierre Cougoul, Jeremie Dion, Lucie Oberic, Anne Huynh, Illias Tazi, Aurore Perrot

**Affiliations:** ^1^ Faculty of Medicine and Pharmacy, Department of Hematology, Mohammed VI University Hospital, Cadi Ayyad University, Marrakesh, Morocco, uca.ma; ^2^ Department of Hematology, Toulouse University Cancer Institute – Oncopole, Toulouse, France; ^3^ Department of Internal Medicine, Toulouse University Cancer Institute – Oncopole, Toulouse, France; ^4^ Department of Hematology, Toulouse University Cancer Institute – Oncopole, University of Toulouse, Toulouse, France

**Keywords:** anti-BCMA, CAR-T cell, hematotoxicity, stem cell boost

## Abstract

Prolonged and refractory hematotoxicity is a severe complication of anti‐BCMA CAR‐T cell therapy, associated with substantial morbidity. When conventional supportive measures (transfusions and hematopoietic growth factors) fail, alternative strategies are urgently needed. We report the case of a 55‐year‐old man who developed persistent cytopenias after idecabtagene vicleucel. Reinfusion of cryopreserved autologous stem cells (stem cell boost), administered without conditioning chemotherapy, resulted in rapid and complete hematopoietic recovery after 14 days. This case highlights the potential of stem cell boost as a salvage approach for prolonged post‐CAR‐T hematotoxicity and underscores the need for prospective multicenter studies to standardize indications, timing, and procedures for this intervention.

## 1. Introduction

Chimeric antigen receptor T‐cell (CAR‐T) therapy has transformed the management of multiple myeloma (MM) and other hematological malignancies [1, 2]. Despite remarkable efficacy, prolonged cytopenias remain a frequent and clinically significant toxicity, reported in up to 64% of patients [3–5]. Proposed mechanisms include cumulative myelotoxicity from prior therapies, inflammatory damage mediated by cytokine release syndrome (CRS), altered bone marrow microenvironment, and underlying clonal hematopoiesis [4–7]. Persistent cytopenia exposes patients to infections, bleeding, transfusion dependence, and impaired quality of life [3, 4]. Supportive measures (transfusion of blood products and administration of hematopoietic growth factors) constitute the mainstay of management but are insufficient in cases of delayed or absent recovery [4, 6, 8].

The “stem cell boost” (SCB), i.e., reinfusion of previously cryopreserved hematopoietic stem cells without conditioning, has emerged as a potential rescue strategy to accelerate hematopoietic recovery [4, 6, 8]. Its application after CAR‐T therapy remains empirical, with limited evidence mainly from retrospective series and anecdotal reports. We describe a patient with plasma cell leukemia who developed persistent hematotoxicity following CAR‐T cell therapy and who achieved full recovery after SCB.

## 2. Case Presentation

A 55‐year‐old male was diagnosed in June 2019 with plasma cell leukemia. Initial cytogenetics showed complex abnormalities, including *1q gain*, multiple *deletions (7p, 8p, 12p, 18p)*, and *t (11;14)*, without *t (4;14)* or *del 17p*. Two NRAS mutations were also identified.

Induction therapy with four cycles of VRD/PAD (bortezomib, lenalidomide, dexamethasone, and doxorubicin) achieved very good partial response (VGPR). Peripheral blood stem cells were collected after cyclophosphamide (3 g/m^2^) + lenograstim, yielding 8.25 × 10^6^ CD34+cells/kg. The patient underwent tandem autologous stem cell transplant (ASCT) in November 2019 and January 2020, achieving stringent complete remission (sCR) with decreasing minimal residual disease (MRD) as assessed by next‐generation sequencing (NGS) (MRD decrease from 4724 × 10^−6^ to 120 × 10^−6^ before and after ASCT). Consolidation with quarterly VRD followed by lenalidomide maintenance was administered until 2022.

In June 2022, biochemical and bone relapse was documented. Daratumumab, carfilzomib, and venetoclax induced VGPR but were discontinued after 18 cycles due to persistent Grade 3‐4 cytopenias (ICATH and T‐ICAHT) [3, 9]. Bone marrow showed 21% plasma cells. Anti‐BCMA CAR‐T therapy with idecabtagene vicleucel was initiated after bridging with pomalidomide, cyclophosphamide, and dexamethasone. The patient had high hematotoxicity risk (CAR‐HEMATOTOX score 6) at the time of infusion.

CAR‐T infusion (August 6, 2024) was complicated by Grade 2 cytokine CRS requiring tocilizumab and dexamethasone. One‐month assessment confirmed cytological remission, but cytopenias persisted.

After 6 months, a new bone marrow assessment, including karyotype, residual disease screening, lymphoid cell immunophenotyping, and viral PCR, came back negative. The patient remained in sCR with low MRD (3.3 × 10^−3^), yet requiring iterative transfusions in red blood cells, platelets and growth factors support (the transfusion is done; on average; every other week, lenograstim if neutrophil count < 0.5 G/L, erythropoietin).

**FIGURE 1:**
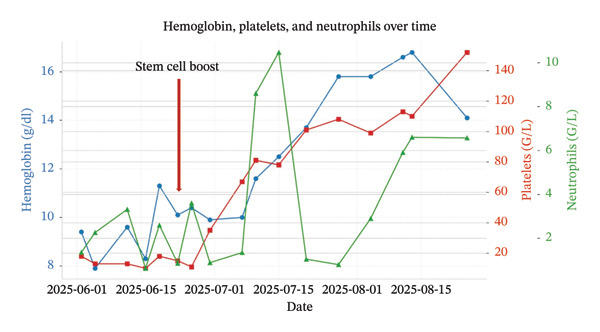
Evolution of cytopenias.

Given refractory cytopenia, a nonconditioned SCB using the residual stem cells initially collected in 2019 (1.81 × 10^6^ CD34+/kg, viability at 87%) was administered on 17 June 2025. Hematopoietic recovery was rapid, with neutrophil and platelet engraftment (neutrophil count > 0.5 G/L and **platelets** count > 20 G/L) at Day 14 and hemoglobin improvement at Day 7 with transfusion independence (Figure 1).

The patient, still in sCR with a normal blood count, continues to be followed up in consultation.

## 3. Discussion

Anti‐BCMA CAR‐T therapy is highly effective in MM but carries substantial risks, particularly prolonged hematotoxicity, which may persist beyond 6 months [1–7]. Proposed mechanisms include cumulative myelotoxicity from prior therapies, inflammatory damage mediated by CRS, altered bone marrow microenvironment, and underlying clonal hematopoiesis [3, 4, 6, 7].

The CAR‐HEMATOTOX score was developed to predict severe hematotoxicity after CAR‐T therapy. Our patient’s score (6) reflected this very high risk. While validated in lymphoma, its extension to MM requires further evaluation [3]. A standard definition of prolonged cytopenias after CAR‐T therapy is missing. Some studies have used ≥ 21 days of Grade 3‐4 cytopenias as a threshold, while others apply different timeframes, such as ≥ 30 days [3, 4, 9]. However, the standardized definitions of ICATH and T‐ICAHT are increasingly being adopted in the literature and in clinical practice [3, 9].

Supportive measures remain the standard management of persistent cytopenias such as thrombopoietin receptor agonists [8, 10]. Sirolimus has been reported to improve cytopenia in a single case [11]. However, SCB is increasingly considered when supportive care fails [4–6, 8]. Reported median CD34+ cell dose is 3.1 × 10^6^/kg (range: 1.7–7.5 × 10^6^/kg) with median time to recovery of 15 days, consistent with our observation. Optimal timing for SCB remains uncertain, ranging from weeks to over 1‐year post‐CAR T [5, 8].

Importantly, because no conditioning regimen is required, this procedure can be safely performed in an outpatient setting (day‐hospital), without the need for prolonged inpatient admission. This characteristic not only improves patient comfort but also confers a highly favorable medico‐economic profile, making SCB an attractive and pragmatic option in eligible cases.

## 4. Conclusion

This case illustrates the feasibility and efficacy of a SCB for refractory prolonged cytopenia after anti‐BCMA CAR‐T therapy. While promising, this strategy requires validation in prospective clinical studies. Beyond its clinical efficacy, the absence of conditioning allowed this intervention to be delivered in a day‐hospital unit, highlighting its excellent cost‐effectiveness profile. Future prospective studies should, therefore, evaluate not only clinical outcomes but also the health‐economic impact of this strategy.

Should stem cell preservation be planned before starting CAR‐T cell therapy?

## Author Contributions

All authors contributed to this work and commented on the manuscript at all stages, and finally, the final version was approved for publication.

## Funding

The author received no specific funding for this work.

## Consent

Consent was obtained from the patient to publish this report in accordance with the journal patient consent policy.

## Conflicts of Interest

The authors declare no conflicts of interest.

## Data Availability

Data sharing is not applicable to this article as no datasets were generated or analyzed during the current study.
